# Hydrolytic Degradation and Mechanical Stability of Poly(ε-Caprolactone)/Reduced Graphene Oxide Membranes as Scaffolds for In Vitro Neural Tissue Regeneration

**DOI:** 10.3390/membranes8010012

**Published:** 2018-03-05

**Authors:** Sandra Sánchez-González, Nazely Diban, Ane Urtiaga

**Affiliations:** Department of Chemical and Biomolecular Engineering, University of Cantabria, Avda. Los Castros s/n, 39005 Santander, Spain; sandra.sanchez@unican.es (S.S.-G.); urtiaga@unican.es (A.U.)

**Keywords:** hydrolytic bulk degradation mechanism, in vitro human neural models, neural tissue regeneration, poly (ε-caprolactone), reduced graphene oxide

## Abstract

The present work studies the functional behavior of novel poly(ε-caprolactone) (PCL) membranes functionalized with reduced graphene oxide (rGO) nanoplatelets under simulated in vitro culture conditions (phosphate buffer solution (PBS) at 37 °C) during 1 year, in order to elucidate their applicability as scaffolds for in vitro neural regeneration. The morphological, chemical, and DSC results demonstrated that high internal porosity of the membranes facilitated water permeation and procured an accelerated hydrolytic degradation throughout the bulk pathway. Therefore, similar molecular weight reduction, from 80 kDa to 33 kDa for the control PCL, and to 27 kDa for PCL/rGO membranes, at the end of the study, was observed. After 1 year of hydrolytic degradation, though monomers coming from the hydrolytic cleavage of PCL diffused towards the PBS medium, the pH was barely affected, and the rGO nanoplatelets mainly remained in the membranes which envisaged low cytotoxic effect. On the other hand, the presence of rGO nanomaterials accelerated the loss of mechanical stability of the membranes. However, it is envisioned that the gradual degradation of the PCL/rGO membranes could facilitate cells infiltration, interconnectivity, and tissue formation.

## 1. Introduction

In the recent years, there has been an increased interest in the functionalization of biocompatible polymers traditionally used for biomedical applications and FDA approved, such as poly(ε-caprolactone) (PCL), in order to incorporate different chemical, mechanical, or electrical stimuli, and therefore converting these polymers from plain cell supports to tissue regenerative inductive materials [1,2]. Different strategies have been used to introduce functional cues into polymers: loading with protein and growth factors [3,4], polymer blending or copolymerization [5,6], and the formation of composites with different types of nanomaterials [7,8,9] are among the most popular approaches. Particularly, while under the shadow of certain controversy, graphene and graphene derivatives, as graphene oxide (GO), have been studied to exploit their peculiar properties: capacity to interact with biomolecules, cells, and tissues, and to enhance the mechanical, electrical, and/or magnetic properties of polymer–graphene composite materials [10,11]. Moreover, graphene has demonstrated the potential to direct differentiation into neural cell lineages of numerous stem cell types, such as embryonic stem cells (ESCs), neural stem cells (NSCs), mesenchymal stem cells (MSCs), and induced pluripotent stem cells (iPSCs) [12].

PCL is a semi-crystalline and hydrophobic aliphatic polyester, biocompatible and bioresorbable. It has received a great attention for biomedical applications as an implantable biomaterial for sutures, wound dressing, and scaffolds for tissue repair [13,14]. In our previous works [15,16], flat membranes of plain PCL, PCL/GO, and PCL/partially reduced graphene oxide (rGO) were developed by a facile phase inversion fabrication method using nontoxic reagents. The membranes showed high porous morphology that provided favorable nutrient transport properties, as well as suitable cell adhesion and proliferation, particularly, PCL with graphene based nanoplatelets. Furthermore, the introduction of rGO into the PCL matrix enhanced the nutrient transport properties, which suggested the increased water wettability of the membranes. Therefore, the PCL/rGO membranes were considered to have great potential to act as scaffolds for neural cells in perfusion bioreactors, in particular, for the regeneration of neural tissue from stem cells from human origin to fabricate in vitro human neural models.

After figuring out the promising properties exhibited by the PCL/rGO membranes and accounting for their use in neural models, the study of the in vitro hydrolytic degradation route and stability behavior of these innovative membranes, as a non-permanent scaffolding material, is crucial. Ideal scaffolds should maintain their properties for sufficient time to complete their function [17]. PCL is a long-term stable polymer when subjected to hydrolytic degradation conditions, and therefore requires 2–4 years for its complete degradation, depending on the starting molecular weight of the PCL [14]. However, it has been widely observed that the incorporation of carbon based nanomaterials into the polymer matrix on the hydrolytic degradation alters the degradability of the polymeric matrix. For instance, Duan et al. [18] observed the enhancement of the wettability of a poly(l-lactide)(PLLA)-GO composite, a behavior that was ascribed to the presence of oxygen-containing groups on the surface of the GO nanoplatelets, facilitated the scission of the polymer macromolecular chains, and consequently, the scaffolds experienced a faster hydrolytic degradation. Similar behavior was observed by Zhao et al. [19], who developed nanocomposites of PLLA with multiwalled carbon nanotubes (MWNTs). Nevertheless, the literature also provides studies showing that the incorporation of graphene nanoplatelets into the polymer matrix produced the opposite effect, i.e., a reduction of the biodegradation rate of the material, due to the hydrophobic constitution of graphene [20,21]. Recently, Murray et al. [21] reported the enzymatic degradation of PCL/rGO mixtures and composites. On the one hand, the presence of rGO below 5 *w/w* % did not significantly influence the enzymatic degradation kinetics. On the other hand, rGO incorporation above 5 *w/w* % in the PCL/rGO composites caused a deceleration of the enzymatic degradation. This was attributed to the higher hydrophobicity of the composite PCL/rGO materials. While enzymatic degradation facilitates the analysis of the degradation changes on the non-permanent polymer devices under acceptable timeframes, long-term hydrolytic studies simulate physiological conditions more adequately. However, it has been demonstrated that accelerated enzymatic studies of PCL scaffolds led to very different degradation mechanisms, and consequently functional properties, than long-term hydrolytic degradation [22]. To the best of our knowledge, the long-term hydrolytic degradation of PCL/graphene composites or blends has not been reported so far.

The aim of this work consisted in the study of the long-term hydrolytic degradation of mixed matrix membranes of PCL/rGO [16]. Also, plain PCL membranes under hydrolytic degradation were evaluated and compared. In vitro conditions were simulated by immersion of the membranes in a phosphate buffer solution (PBS, pH 7.4) at 37 °C. The evolution of the functional, morphological, chemical, and thermal characteristics of the PCL/rGO membranes was evaluated during a period of 1 year. A degradation kinetics and hydrolytic pathway of the membranes were proposed and their structural stability was analyzed. Additionally, the degradation products during the study were monitored in order to elucidate potential effects on cell cytotoxicity.

## 2. Materials and Methods

### 2.1. Membrane Preparation

PCL pellets (average molecular weight, 80 kDa; Sigma-Aldrich, Madrid, Spain) were used to fabricate PCL/rGO membranes using a phase inversion technique. The synthesis of rGO particles adapted from Ribao et al. [23], as well as the fabrication of the membranes, was described in detail in our previous work [16]. Control PCL membranes without rGO nanoplatelets were also prepared for comparison.

Hydrolytic degradation experiments were performed on PCL/rGO and PCL membranes working under simulated in vitro bioreactor conditions. A sufficient number of membranes were submerged in a phosphate buffer solution (PBS, pH 7.4) and placed in an incubator at 37 °C. Separate solutions were used for testing PCL/rGO and PCL membranes. Samples were taken out of the solution for characterization at predetermined degradation time intervals: 0, 2, 4, 6, 9, and 12 months. PBS was prepared as follows: 8 g of NaCl, 0.2 g of KCl, 1.44 g of Na_2_HPO_4_, and 0.24 g of KH_2_PO_4_ were solubilized in 800 mL of distilled water. Then, the pH was adjusted to 7.4 with HCl (0.1 mol/L) and made up to 1 L with distilled water. PBS was autoclaved for sterilization. The membranes were sterilized by immersion in ethanol/water 70/30 *v/v* % and subsequent exposure to UV light for 20 min in a laminar cabinet.

### 2.2. Characterization

#### 2.2.1. Functional Properties

Axial tensile tests of the membranes were done using a servo-hydraulic testing universal machine (ME-400, SERVOSIS, Madrid, Spain) following the ISO standard for thin plastic membranes (ASTM D882-12). The specimens had an area of 40 × 6 mm^2^, and the tests were carried out using a load cell of 1.25 kN at a constant elongation speed of 8 mm/s.

A tangential flow filtration system was used to characterize the flux of nutrients across the membranes. The cross-flow filtration set up was already defined in our previous work [16]. A model feed solution was prepared, consisting of protein bovine serum albumin (BSA, >96% purity, Sigma-Aldrich) at 0.4 g/L in PBS (pH 7.4). The membrane was previously stabilized with ultrapure (UP) water at 0.1 bar for 1 h. Afterwards, the BSA model solution was circulated throughout the feed compartment of the membrane cell, and a transmembrane pressure of 0.1 bar was applied during 4 h of operation. The permeate solution was collected and weighed while the retentate was recirculated to the feed tank. The change with time (t) of total BSA solution flux ($J_{T}\;$(L·m^−2^·h^−1^)) was determined as

(1) $$J_{T}=\left(W_{T,permeated}\times \rho_{PBS,37{}^{\circ}C}\right)/\left(\Delta t\times A_{e}\right)$$

where $W_{T,permeated}\;\left(g\right)$ is the mass of permeate collected in the time interval *Δt* (h) and using an effective surface area *A_e_* (m^2^), and $\rho_{PBS,37{}^{\circ}C}$ (g·L^−1^) the PBS density at 37 °C. At least two membrane replicates were analyzed for each degradation time.

#### 2.2.2. Physical–Chemical Properties

The average molecular weight of the membranes was determined by gel permeation chromatography (GPC model 510, Waters, Madrid, Spain). Three size exclusion chromatography columns of styrene divinyl benzene copolymer were placed in series (model Styragel HR 5E, Waters) and a refractometer (model 410, Waters) was used for detection. The columns were thermostatized at 40 °C, and the measurements were carried out using 1 mL/min of tetrahydrofuran (THF 99.9%, Panreac, Barcelona, Spain) as carrier. Membrane samples were solubilized in THF at a concentration of 0.5 mg/mL. PCL/rGO in THF samples were centrifuged for 1 h and filtered through a 0.45 µm filter before GPC injection, in order to avoid rGO contamination of the GPC columns. The values of molecular weight distribution were obtained by the Empower 2 software (Waters). The molecular weights were determined using a universal calibration curve related to polystyrene standards (Shodex, Waters, Cerdanyola del Vallès, Spain) corrected by the Mark–Houwink–Sakura equation and the corresponding PCL coefficients. Measurements were done in duplicate.

Thermal properties of the samples at 0 and 12 months of degradation were evaluated by differential scanning calorimetry (DSC, DSC-131, SETARAM Instrumentation, Caluire, France) at a scan rate of 10 °C/min. Samples (5–10 mg) were heated from room temperature to 100 °C (first heating run). After 10 min stabilization at 100 °C, the samples were cooled down to 0 °C (cooling run) and finally heated up again to 100 °C (second heating run) after stabilization. The degree of crystallinity, $\chi_{C}$ (%) was calculated using Equation (2) [24], where $\Delta H_{m}^{0}$ (J·g^−1^) is the melting enthalpy calculated from the second heating ramp, $\Delta H_{m}^{0}$ is the melting enthalpy for a 100% crystalline PCL (139.5 J·g^−1^ [24]) and is the mass fraction of rGO in the PCL membrane.

(2) $$\chi_{C}=\Delta H_{m}/\left[\left(1-\beta \right)\times \Delta H_{m}^{0}\right]$$

The concentration of 6-hydroxycaproic acid (6-HCA), typically found as monomer degradation product of PCL, was analyzed in the PBS medium where the membranes were submerged. The UV–vis spectrophotometer (UV-1800 model, Shimadzu, Duisburg, Germany) was set at a 210 nm wavelength [25]. Measurements of 6-HCA were carried out after 6 and 12 months of degradation time. The presence of rGO nanoplatelets on the PBS medium and in the membrane matrix after 12 months of degradation was analyzed (see Supplementary Materials).

Microscopic images of the membranes were obtained using a scanning electron microscope (SEM, EVO MA 15, Carl Zeiss, Madrid, Spain) at a voltage of 20 kV, in order to determine the structure and morphology of the surface and cross section of the membranes. Samples for the cross-section images were frozen in liquid nitrogen to be fractured. All the samples were kept overnight at 30 °C under vacuum, and were gold sputtered before examination. Moreover, visual inspection of the membrane was recorded by taking photographs of the same membrane specimen periodically.

Before any testing, membranes were cleaned with UP water to remove any possible salt deposit. Results are expressed as average ± standard deviation.

## 3. Results

### 3.1. Functional Properties

**Figure 1:**
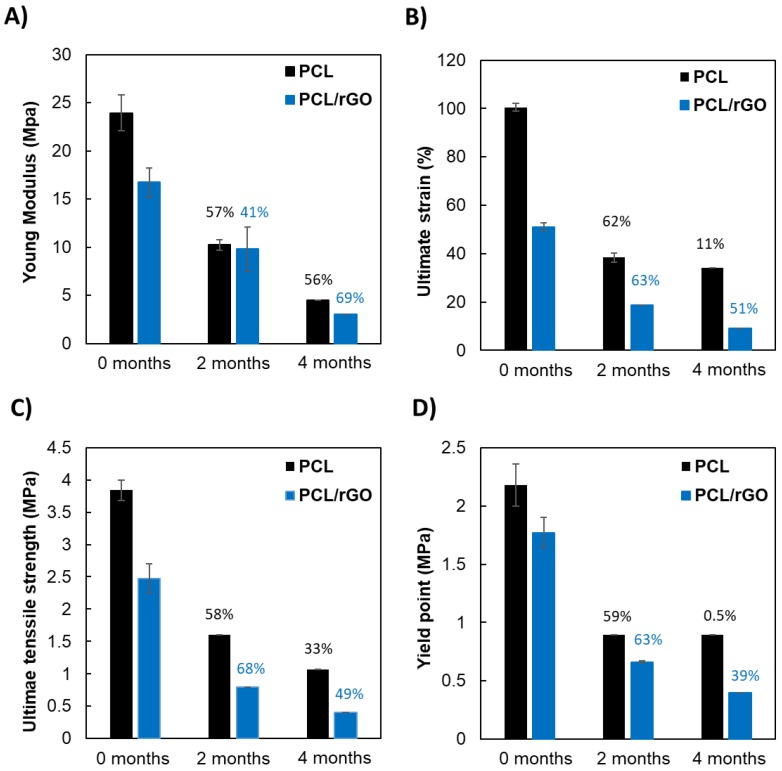
Mechanical properties of poly(ε-caprolactone) (PCL) and PCL/reduced graphene oxide (rGO) membranes. (**A**) ultimate tensile stress; (**B**) Young modulus; (**C**) ultimate strain; and (**D**) yield point for PCL and PCL/rGO membranes at 0, 2, and 4 months of degradation. (% values represent the reduction of the mechanical parameters between degradation times.)

Figure 1 shows the mechanical properties of PCL and PCL/rGO membranes during the degradation study. Figure 1A–D shows the Young modulus, yield point, ultimate tensile strength, and ultimate strain, respectively. Mechanical tests were not feasible beyond 4 months, due to the loss of mechanical stability. Specifically, the PCL membranes could be handled until 6 months, while the PCL/rGO membranes could not be manipulated after 4 months. Videos confirming the poor stability of the PCL and PCL/rGO membranes after 12 months of immersion in PBS are included in the Supplementary Materials (Videos S1 and S2). During the hydrolytic degradation study, a gradual reduction in the mechanical properties of both membranes was observed in the values of mechanical parameters (Figure 1). Overall, at time 0, the presence of rGO in the polymer matrix significantly reduced the mechanical properties in comparison to the plain PCL membranes. After 2 months, PCL and PCL/rGO membranes showed homogeneous reduction of mechanical properties. For instance, PCL membranes showed a reduction of mechanical properties in the range 57–62%, in a narrow range for all the properties evaluated. The reduction of properties for PCL/rGO membranes was also encountered, mainly around 63–68%, with the exception of the Young Modulus that suffered a 41% drop. After 4 months, PCL/rGO still suffered similar reduction of mechanical properties as in previous degradation times, while PCL membranes presented more disordered behavior: for instance, the yield point barely changed while the Young modulus showed a 60% reduction.

**Figure 2:**
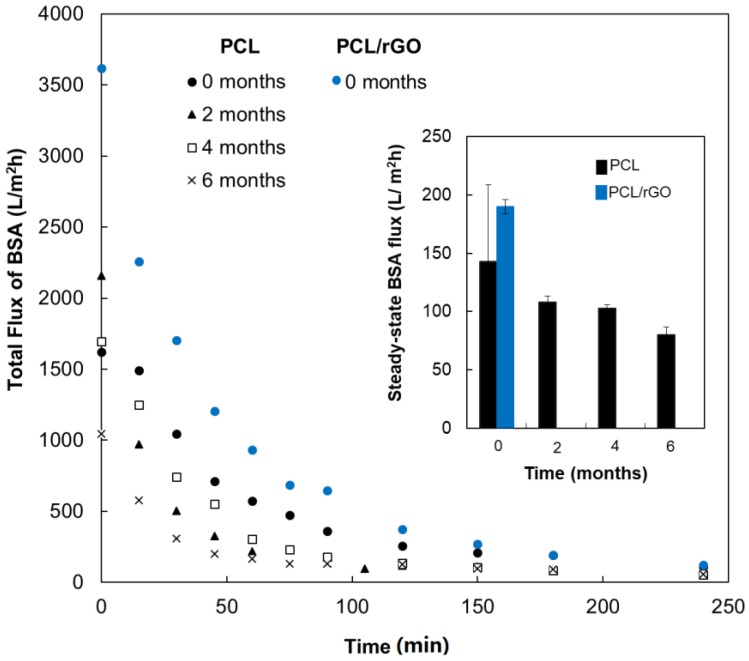
Total flux decay of BSA model solution of average values of PCL membranes during 240 min at different times of degradation. Flux data for PCL/rGO membrane at *t* = 0 months were also included (deviation bars not shown for the shake of clarity). Inset shows the values of BSA solution flux at steady state for PCL and PCL/rGO membranes at 0, 2, 4, and 6 months of degradation.

Figure 2 plots the change with the filtration time of the volumetric flux of BSA model solution through the PCL membranes for specimens that had been submerged in the hydrolytic bath for 0, 2, 4, and 6 months, and PCL/rGO only at *t* = 0 months. BSA permeation tests for PCL/rGO membranes after in vitro degradation (*t* > 0 months) could not be performed because they could not withstand the transmembrane pressure of the filtration device. The membranes experienced a sharp flux drop during the first 2 h of filtration, with a reduction of 88.1 ± 2.9% in each point of degradation. Afterwards, it could be assumed that the flux reached a pseudo steady state (Inset of Figure 2). The BSA solution fluxes at this steady state were as follows: 143 ± 66 > 108 ± 5 > 103 ±3 > 80 ± 7 L·m^−2^·h^−1^ at 0, 2, 4, and 6 months of degradation, respectively. Similarly, the BSA solution flux through PCL/rGO membranes at 0 months decayed from the initial value of 3620 ± 356 L·m^−2^·h^−1^ to a stable flux of 190 ± 68 L·m^−2^·h^−1^ (drop of 94.5 ± 2.4%).

### 3.2. Physical–Chemical Properties Characterization

**Figure 3:**
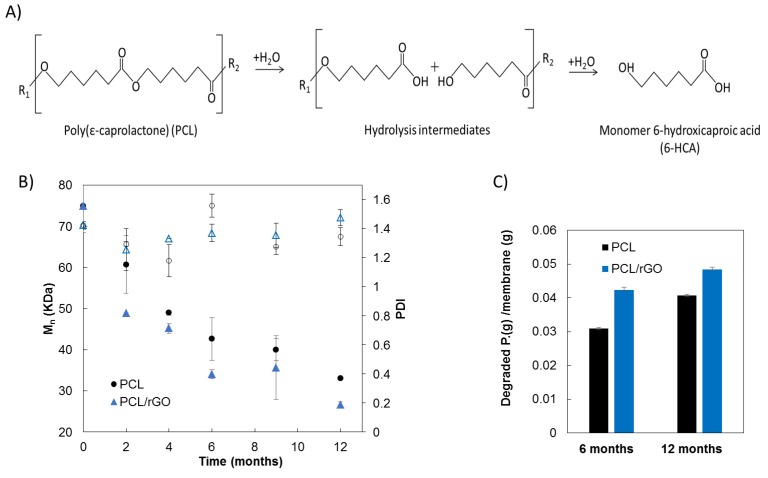
(**A**) Scheme of the PCL hydrolytic degradation process, adapted from Woodruff et al. [14]; (**B**) Change of the number average molecular weight (*M_n_*, filled symbols) and polydispersity index (PDI, empty symbols); and (**C**) mass of degradation product 6-HCA in the PBS formed during degradation process of PCL and PCL/rGO membranes.

Figure 3A shows the hydrolysis degradation pathway of the PCL polymer. The cleavage of the ester bonds of PCL is produced upon the reaction with water, forming carboxyl end-groups, and the progressive reduction of the average molecular size to give water-soluble degradation products, including oligomers and monomer (6-HCA), that diffused out of the membrane matrix and solubilized in the PBS medium [26].

The progress of the number average molecular weight (*M_n_*) with the degradation time is presented in Figure 3B. Both PCL and PCL/rGO membranes showed a progressive decrease in *M_n_*. After two months of degradation, the *M_n_* of the membranes suffered a significant reduction from the initial value of 75 ± 6 kDa to 61 ± 7 kDa for PCL membranes (drop of 19%), and to 49 kDa for PCL/rGO membranes (drop of 35%). At 12 months, *M_n_* decreased further to 33 ± 0.04 kDa (56%) for PCL membranes, and to 27 ± 0.75 kDa (65%) for PCL/rGO membranes. The polydispersity index (PDI) of the molecular weight distribution remained almost constant during the degradation period, i.e., PDI values of 1.42 at *t* = 0 months and 1.47 at *t* = 12 months for PCL films, and 1.34 at *t* = 12 months for PCL/rGO films were obtained (Figure 3B). Regarding the hydrolysis kinetics of polymers, it usually follows second order reaction kinetics, that is, the rate of the reaction is proportional to the concentration of water and the concentration of chemical bonds susceptible of hydrolysis, i.e., carboxylic bonds for polyesters [27] (see Equations (S1) and (S2) in Supplementary Materials). Figure S1 (Supplementary Materials) shows good agreement of the fitting of the experimental data to 1/*M_n_* vs *t*, which indicates that the hydrolysis of our membranes proceeded according to second order kinetics. Moreover, in Figure S1, it was shown that the hydrolysis kinetics of PCL/rGO membranes was only slightly faster (approximately 1.4 times) than the hydrolytic kinetics of PCL membranes.

Figure 3C illustrates the concentration of 6-HCA per unit mass of membrane released to the PBS medium after 6 and 12 months of hydrolytic degradation. The concentration of the 6-HCA in the PBS increased significantly with the degradation time, as expected. The 6-HCA concentration in the PBS medium was higher for PCL/rGO than for PCL membranes, in good agreement with the evolution of the molecular size observed in Figure 3B. The presence of 6-HCA in the buffer solutions barely affected the pH (data not shown). During the degradation test, an attempt to evaluate what happened with the rGO nanoplatelets of the PCL/rGO membranes was done. After re-dissolving the PCL/rGO membranes in THF, the presence of rGO nanoplatelets in the membranes was visually confirmed at 0 months and after 12 months of degradation (see Figure S2 of the Supplementary Materials). It can also be appreciated that the precipitated rGO was qualitatively more abundant (higher mass concentration) in the samples corresponding to 12 months of degradation than in the PCL/rGO membranes at *t* = 0. Moreover, the UV–vis spectrum of the PBS that contained the PCL/rGO membranes during 12 months of degradation did not show the rGO representative peak around 270 nm [28]. These qualitative results led us to think that rGO mainly remained in the solid material of the PCL/rGO membranes.

**Figure 4:**
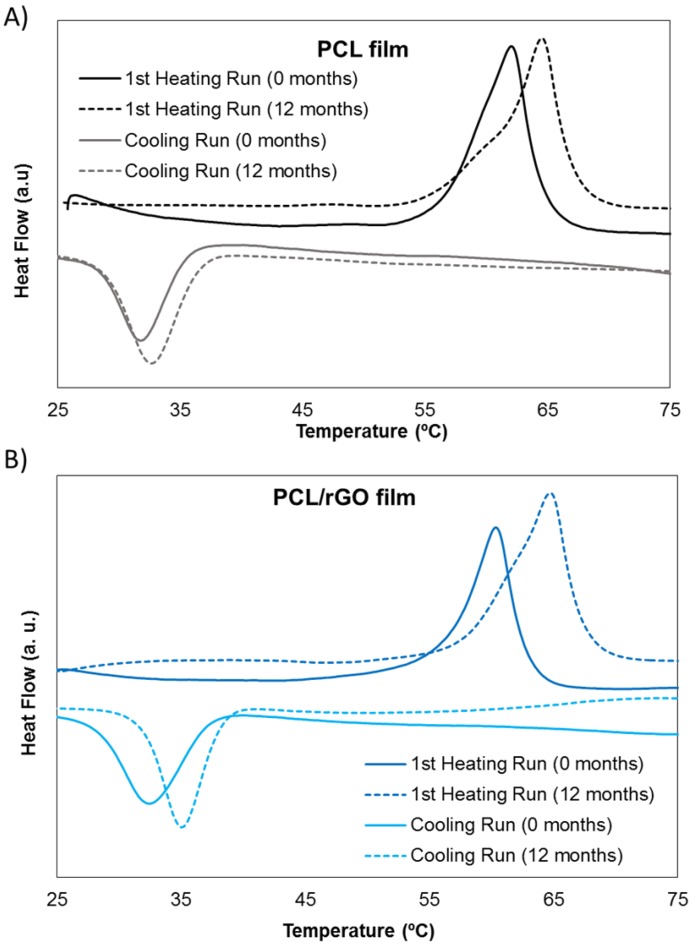
DSC thermogram of PCL (**A**) and PCL/rGO (**B**); membranes at 0 and 12 months of degradation.

Furthermore, Figure 4 represents the DSC thermograms of the PCL (A) and PCL/rGO membranes (B) at 0 and 12 months. The initial value of the melting temperature (*T_m_*) (0 months) was 62.10 °C for PCL membranes and 60.36 °C for PCL/rGO membranes. After 12 months of degradation time, *T_m_* increased to 64.54 °C for PCL and to 64.72 °C for PCL/rGO membranes. The crystallization temperature, *T_c_*, increased as well during the degradation period in both types of membranes, from 31.75 °C to 32.63 °C for PCL, and from 32.45 °C to 35.00 °C for PCL/rGO. The initial $\chi_{C}$ of PCL/rGO was 41%, and increased to 46% after 12 months. Meanwhile, PCL membranes crystallinity varied from 35% to 44%. The higher crystallinity of the degraded samples pointed to the preferential hydrolytic attack of the amorphous polymer phase.

**Figure 5:**
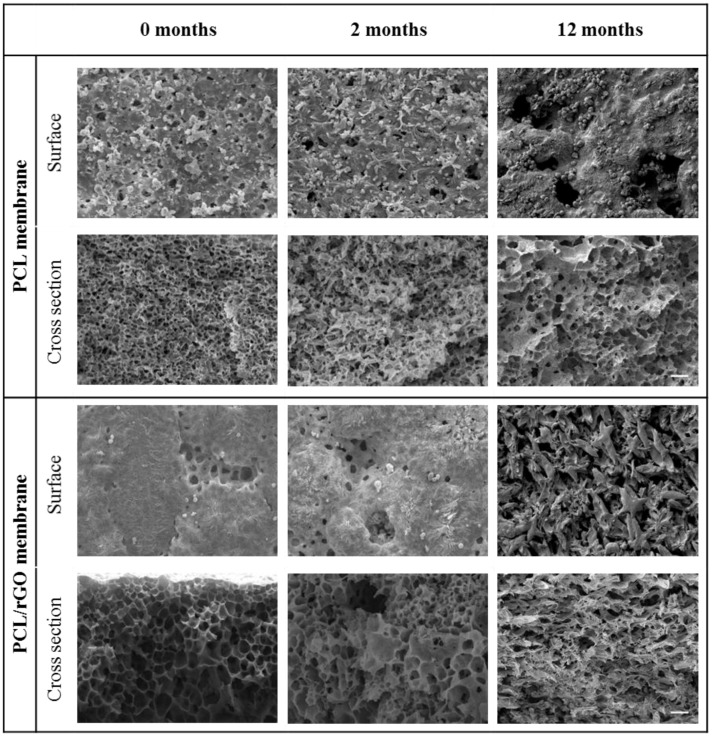
SEM images of PCL and PCL/rGO membranes at 0, 2, and 12 months of hydrolytic degradation. The scale bars represent 10 µm.

The membranes did not suffer any significant reduction in dimensions (width, length, and thickness) during the degradation time (Figure S3, Supplementary Materials). Regarding the microscopic morphology, SEM images of the surface and cross section of the PCL and PCL/rGO membranes at 0, 2, and 12 months of hydrolytic degradation are illustrated in Figure 5. Overall, a noticeable change in the morphology of the membranes can be observed. The surface of both PCL and PCL/rGO membranes eroded slightly from 0 to 2 months, and then very notably after 12 months of degradation. The morphological degradation of the internal structure of the membranes was also evident, as shown in the SEM cross section images.

## 4. Discussion

PCL/rGO and control PCL membranes fabricated in this work degraded continuously under the presence of PBS simulating in vitro culture conditions. The molecular weight presented a progressive reduction (Figure 3B), produced by the hydrolytic chain scission of the ester group due to water penetration. The degradation kinetics of our membranes corresponded to second order kinetics, in agreement with typical hydrolysis of large molecular weight polyesters [27]. Also, the maintenance of PDI values (Figure 3B) during the degradation period indicated that all the carboxylic bonds of the polymer chain had equal reactivity, in agreement with the obtained second order kinetics. In spite of the hydrophobic character of the rGO nanoplatelets [10], a slight, though not significant, acceleration of the hydrolytic degradation for PCL/rGO membrane was observed in comparison to the plain PCL membranes in terms of molecular weight change. The monomer 6-HCA, as the main indicator of degradation products, was released and diffused into the buffer media (Figure 3C). Therefore, its concentration progressively increased with the degradation period, and showed slightly higher values for buffer media containing PCL/rGO membranes than for PCL membranes, in agreement with the results of the molecular weight degradation kinetics. Moreover, the higher degree of crystallinity, as well as the increase on the thermal properties after 12 months of degradation, pointed to the preferential hydrolytic attack of the polymer amorphous region in both membranes [17]. Finally, the internal morphology of the membranes suffered a clear change (Figure 5), while the dimensions of the membranes remained constant during the degradation process (Figure S3). According to the aforementioned results, in the present work, degradation of the membranes proceeded via bulk degradation mechanism [29]. Bulk degradation mechanism of PCL networks was also reported by [22] under similar hydrolytic degradation conditions. The similar tendencies observed in the present work on the molecular weight reduction for PCL and PCL/rGO membranes, points to the high porosity of the fabricated membranes as the main cause for the bulk hydrolysis mechanism. The porous internal morphology favored the water penetration and the outward diffusion of the degradation products [30]. In comparison to other reported works with similar molecular weight (PCL 80 kDa) [31,32], our fabricated membranes demonstrated an accelerated degradation rate.

All mid- and end-point degradation products must be thoroughly investigated for possible immunogenic reactions [33]. During the progress of the polymer degradation, it was observed that the rGO nanoplatelets remained mainly in the membrane. This was consistent with the results of Murray et al. [21]. They also reported that PCL/rGO blended materials increased the relative concentration of rGO during enzymatic degradation from 5 *w/w* % to 19 *w/w* %, and did not observe cytotoxicity on L-929 fibroblast cells growing for short periods. In our previous work [16], we also observed a positive biocompatibility on glioblastoma cells of the PCL membranes containing rGO nanomaterials after 14 days of culture. These null cytotoxic results are also in agreement with the low pH acidification of the buffer solution observed in our system (results not shown) that would not likely turn into a negative cellular response [34].

The presence of rGO nanoplatelets in the polymer matrix significantly reduced the mechanical integrity of the membranes at any degradation time. This effect was attributed to a restriction of the mobility of the polymer chains [24], and to defects and gaps created by the presence of rGO in the polymer matrix [35]. Also, the presence of rGO caused a faster and more intense loss of mechanical properties and structural stability for PCL/rGO membranes in contrast to PCL membranes (Figure 1 and Videos S1 and S2) that could also be explained by the same causes that decreased the initial mechanical properties of PCL/rGO in contrast to PCL membranes, as previously explained. Despite the fast loss of mechanical properties of the PCL/rGO membranes, after 4 months of degradation, these materials still comply sufficiently with the mechanical properties required for materials to sustain neural tissues. Actually, the mechanical stiffness of the 4-month degraded membranes are closer to the values of the hydrogels typically employed as scaffold materials for neural tissue regeneration, i.e., Matrigel [36], modified gelatin [37], polyethylene glycol, or alginate hydrogels [38,39]. For instance, a broad range of Young modulus values, i.e., in the order of 0.2–20 kPa for alginate hydrogel 40 and 0.1–1.2 MPa for modified gelatin [37], can be found for these materials. Nevertheless, although Matrigel is one of the most employed materials for scaffolds in neural adhesion and proliferation, its relatively weak mechanical strength and significant degradation over long-term culture has been considered a drawback for its use in in vitro neural models [40]. Apart from the effect of the membrane stiffness on the induction of mechanical cues over the cells, it is envisaged that the mechanical properties loss of the material could be substituted by the tissue mechanical stability if there is an equivalent rate of membrane structural disintegration and tissue regeneration. In the field of neural tissue regeneration, in vitro models of cerebral organoids require only 8–10 days for the appearance of neural identity and 20–30 days for the formation of defined brain regions [36]. Mahoney and Anseth [39] confirmed the suitable use of polyethylene glycol hydrogels to act as cell carriers for transplantation into the central nervous system (CNS), with an accelerated loss of mechanical properties in 12 days. In general, an adequate scaffold material should lose mechanical properties at an approximate rate of 8%/week during in vivo degradation [41], and neural scaffold materials would ideally degrade over a period of 2–8 weeks via hydrolysis, ion exchange, or through enzymatic reactions [33]. All the previous works support the idea that the rate of neural tissue regeneration could be comparable to the degradation rate behavior of our PCL and mainly to PCL/rGO membranes. Actually, preliminary experiments on neural progenitor cells (NPC) differentiation and maturation have been performed for 20 days on PCL and PCL/rGO membranes, showing promising cell coverage (unpublished data) and adequate structural integrity for the manipulation.

Regarding the behavior of the BSA solution flux through the membranes, the initial pronounced reduction of the flux (Figure 2) was associated with the internal fouling due to BSA protein adhesion to the pore walls [16]. The progressive decrease of the steady-state BSA fluxes for PCL membranes during the degradation time could be attributed to the gradual loss of structural integrity under hydrodynamic pressure, causing the membrane compaction, and therefore, the pore size reduction during filtration assays [30]. Regardless, the significant reduction of the nutrient flux at steady state, PCL and PCL/rGO membranes still displayed a comparable total BSA solution flux to that reported by Bettahalli et al. [42] for commercial poly(ether-sulfone) hollow fibers, theoretically sufficient to supply the needs of glucose consumption to more than three layers of cells under confluence in a perfusion bioreactor.

## 5. Conclusions

The present work reports on the evaluation of the hydrolytic degradation of novel PCL/rGO porous membranes fabricated by phase inversion technique. The hydrolytic degradation during a long term period of 12 months of these PCL/rGO membranes was evaluated in this work, in order to study the membrane capacity to act as scaffold for in in vitro bioreactors for neural tissue regeneration and its further use as in vitro human neural models.

Both, PLC/rGO membranes and PCL membranes (control membranes) exhibited a fast degradation rate. This work demonstrates that the high porous membrane structure obtained as a result of the phase inversion manufacturing technique was the main factor on the acceleration of the degradation, as it could promote water penetration, and therefore facilitate the bulk hydrolytic mechanism of the membranes. The molecular weight decreased, following second order kinetic rate, characteristic of these types of polyesters of large molecular weight. As a result, there was a loss of the membrane’s mechanical resistance, an enhancement of the crystallinity, and the formation of PCL degradation products, such as the monomer 6-hydroxycaproic acid, released to the hydrolytic media. Besides the aforementioned alterations, the changes in the porous morphology without any observable modification of the sample dimensions led to the conclusion that degradation proceeded via bulk hydrolysis mechanism. The introduction of rGO nanoplatelets into the PCL matrix only slightly accelerated the degradation rate. Particularly, the presence of rGO reduced significantly the mechanical stability of the membranes at all degradation times. However, PCL/rGO membranes still procured sufficient mechanical properties to theoretically comply with the specifications of the neural tissue regeneration. Besides, the degradation rate of the membranes herein reported would perfectly fit the rate of neural tissue regeneration that would need around 1 month to be completed. The rGO nanoplatelets remained preferentially in the polymer matrix of the membrane during the degradation process and, according to previous works, the degradation products of similar PCL/rGO blended materials should not alter the cytotoxicity of the buffer solution. The high porosity that induces exceptional BSA solution flux let us deem that PCL/rGO membranes would be promising candidates to be used as scaffolds for neural tissue regeneration in perfusion bioreactors. Finally, it has to be remarked that experiments to evaluate the performance of the PCL/rGO membranes on dynamic neural cell culture, as well as the assessment of the potential of PCL/rGO membranes to induce stem cell differentiation into neural tissue, are currently under progress.

## Supplementary Materials

The following are available online at http://www.mdpi.com/2077-0375/8/1/12/s1, Video S1: Mechanical behavior of PCL membranes after 12 months of hydrolytic degradation; Video S2: Mechanical behavior of PCL/rGO membranes after 12 months of hydrolytic degradation; Equations (S1) and (S2) demonstrating simplifications and fitting of the experimental data of change of the polymer molecular weight with degradation time to a 2nd order hydrolysis kinetics and Figure S1: Kinetics of the hydrolysis of PCL and PCL/rGO membranes. Fitting of the molecular weight to the second order hydrolysis kinetics (Equation (S2)) is depicted (dotted lines). Figure S2: Photographs showing the rGO content at 0 and 12 months of degradation of the PCL/rGO membranes. Figure S3: Photographs showing the visual aspect of the wet PCL and PCL/rGO membranes at 0 and 12 months of degradation.

## References

[1] Guo B., Lei B., Li P., Ma P.X. (2015). Functionalized scaffolds to enhance tissue regeneration. Regen. Biomater..

[2] Liu X., Holzwarth J.M., Ma P.X. (2012). Functionalized Synthetic Biodegradable Polymer Scaffolds for Tissue Engineering. Macromol. Biosci..

[3] Kim Y.H., Jyoti M.A., Song H.Y. (2014). Immobilization of cross linked Col-I-OPN bone matrix protein on aminolysed PCL surfaces enhances initial biocompatibility of human adipogenic mesenchymal stem cells (hADMSC). Appl. Surf. Sci..

[4] Wang Z., Sun B., Zhang M., Ou L., Che Y., Zhang J., Kong D. (2012). Functionalization of electrospun poly(ε-caprolactone) scaffold with heparin and vascular endothelial growth factors for potential application as vascular grafts. J. Bioact. Compat. Polym..

[5] Patrício T., Domingos M., Gloria A., Bártolo P. (2013). Characterisation of PCL and PCL/PLA scaffolds for tissue engineering. Procedia CIRP.

[6] Pêgo A.P., Poot A.A., Grijpma D.W., Feijen J. (2001). Copolymers of trimethylene carbonate and epsilon-caprolactone for porous nerve guides: Synthesis and properties. J. Biomater. Sci. Polym. Ed..

[7] Koupaei N., Karkhaneh A. (2016). Porous crosslinked polycaprolactone hydroxyapatite networks for bone tissue engineering. Tissue Eng. Regen. Med..

[8] Augustine R., Dominic E.A., Reju I., Kaimal B., Kalarikkal N., Thomas S. (2014). Electrospun polycaprolactone membranes incorporated with ZnO nanoparticles as skin substitutes with enhanced fibroblast proliferation and wound healing. RSC Adv..

[9] Crowder S.W., Liang Y., Rath R., Park A.M., Maltais S., Pintauro P.N., Hofmeister W., Lim C.C., Wang X., Sung H.-J. (2013). Poly (ε-caprolactone)-carbon nanotube composite scaffolds for enhanced cardiac differentiation of human mesenchymal stem cells. Nanomedicine.

[10] Goenka S., Sant V., Sant S. (2014). Graphene-based nanomaterials for drug delivery and tissue engineering. J. Control. Release.

[11] Shin S.R., Li Y.-C., Jang H.L., Khoshakhlagh P., Akbari M., Nasajpour A., Zhang Y.S., Tamayol A., Khademhosseini A. (2016). Graphene-based materials for tissue engineering. Adv. Drug Deliv. Rev..

[12] Bressan E., Ferroni L., Gardin C., Sbricoli L., Gobbato L., Ludovichetti F., Tocco I., Carraro A., Piattelli A., Zavan B. (2014). Graphene based scaffolds effects on stem cells commitment. J. Transl. Med..

[13] Mondal D., Griffith M., Venkatraman S.S. (2016). Polycaprolactone-based biomaterials for tissue engineering and drug delivery: Current scenario and challenges. Int. J. Polym. Mater. Polym. Biomater..

[14] Woodruff M.A., Hutmacher D.W. (2010). The return of a forgotten polymer—Polycaprolactone in the 21st century. Prog. Polym. Sci..

[15] Diban N., Ramos-Vivas J., Remuzgo-Martinez S., Ortiz I., Urtiaga A. (2014). Poly (ε-caprolactone) films with favourable properties for neural cell growth. Curr. Top. Med. Chem..

[16] Diban N., Sanchez-Gonzalez S., Lázaro-Díez M., Ramos-Vivas J., Urtiaga A. (2017). Facile fabrication of poly(ε-caprolactone)/graphene oxide membranes for bioreactors in tissue engineering. J. Membr. Sci..

[17] Bosworth L.A., Downes S. (2010). Physicochemical characterisation of degrading polycaprolactone scaffolds. Polym. Degrad. Stab..

[18] Duan J., Xie Y., Yang J., Huang T., Zhang N., Wang Y., Zhang J. (2016). Graphene oxide induced hydrolytic degradation behavior changes of poly(l-lactide) in different mediums. Polym. Test..

[19] Zhao Y., Qiu Z., Yang W. (2009). Effect of multi-walled carbon nanotubes on the crystallization and hydrolytic degradation of biodegradable poly(l-lactide). Compos. Sci. Technol..

[20] Finniss A., Agarwal S., Gupta R. (2016). Retarding hydrolytic degradation of polylactic acid: Effect of induced crystallinity and graphene addition. J. Appl. Polym. Sci..

[21] Murray E., Thompson B.C., Sayyar S., Wallace G.G. (2015). Enzymatic degradation of graphene/polycaprolactone materials for tissue engineering. Polym. Degrad. Stab..

[22] Castilla-Cortázar I., Más-Estellés J., Meseguer-Dueñas J.M., Escobar Ivirico J.L., Marí B., Vidaurre A. (2012). Hydrolytic and enzymatic degradation of a poly(ε-caprolactone) network. Polym. Degrad. Stab..

[23] Ribao P., Rivero M.J., Ortiz I. (2016). TiO_2_ structures doped with noble metals and/or graphene oxide to improve the photocatalytic degradation of dichloroacetic acid. Environ. Sci. Pollut. Res..

[24] Wang G.S., Wei Z.Y., Sang L., Chen G.Y., Zhang W.X., Dong X.F., Qi M. (2013). Morphology, crystallization and mechanical properties of poly(e-caprolactone)/graphene oxide nanocomposites. Chin. J. Polym. Sci..

[25] Hafeman A.E., Zienkiewicz K.J., Zachman A.L., Sung H.-J., Nanney L.B., Davidson J.M., Guelcher S.A. (2011). Characterization of the Degradation Mechanisms of Lysine-derived Aliphatic Poly(ester urethane) Scaffolds. Biomaterials.

[26] Bölgen N., Menceloglu Y.Z., Acatay K., Vargel I., Piskin E. (2005). In vitro and in vivo degradation of non-woven materials made of poly(ε-caprolactone) nanofibers prepared by electrospinning under different conditions. J. Biomater. Sci. Polym. Ed..

[27] Lyu S., Untereker D. (2009). Degradability of polymers for implantable biomedical devices. Int. J. Mol. Sci..

[28] Krishnamoorthy K., Veerapandian M., Zhang L.H., Yun K., Kim S.J. (2012). Antibacterial efficiency of graphene nanosheets against pathogenic bacteria via lipid peroxidation. J. Phys. Chem. C.

[29] Von Burkersroda F., Schedl L., Göpferich A. (2002). Why degradable polymers undergo surface erosion or bulk erosion. Biomaterials.

[30] Zhang Q., Jiang Y., Zhang Y., Ye Z., Tan W., Lang M. (2013). Effect of porosity on long-term degradation of poly (ε-caprolactone) scaffolds and their cellular response. Polym. Degrad. Stab..

[31] Höglund A., Hakkarainen M., Albertsson A.-C. (2007). Degradation profile of poly(ε-caprolactone)—The influence of macroscopic and macromolecular biomaterial design. J. Macromol. Sci. Part A Pure Appl. Chem..

[32] Lam C.X.F., Savalani M.M., Teoh S.-H., Hutmacher D.W. (2008). Dynamics of in vitro polymer degradation of polycaprolactone-based scaffolds: Accelerated versus simulated physiological conditions. Biomed. Mater..

[33] Thomas M., Willerth S.M. (2017). 3-D Bioprinting of Neural Tissue for Applications in Cell Therapy and Drug Screening. Front. Bioeng. Biotechnol..

[34] Göpferich A. (1996). Mechanisms of polymer degradation and erosion. Biomaterials.

[35] Jin T.X., Liu C., Zhou M., Chai S.G., Chen F., Fu Q. (2015). Crystallization, mechanical performance and hydrolytic degradation of poly(butylene succinate)/graphene oxide nanocomposites obtained via in situ polymerization. Compos. Part A.

[36] Lancaster M.A., Renner M., Martin C.-A., Wenzel D., Bicknell L., Hurles M., Homfray T., Penninger J.M., Jackson A.P., Knoblich J.A. (2013). Cerebral organoids model human brain development and microcephaly. Nature.

[37] Binan L., Tendey C., De Crescenzo G., El Ayoubi R., Ajji A., Jolicoeur M. (2014). Differentiation of neuronal stem cells into motor neurons using electrospun poly-l-lactic acid/gelatin scaffold. Biomaterials..

[38] Banerjee A., Arha M., Choudhary S., Ashton R.S., Bhatia S.R., Schaffer D.V., Kane R.S. (2009). The influence of hydrogel modulus on the proliferation and differentiation of encapsulated neural stem cells. Biomaterials.

[39] Mahoney M.J., Anseth K.S. (2006). Three-dimensional growth and function of neural tissue in degradable polyethylene glycol hydrogels. Biomaterials.

[40] Wan X., Ball S., Willenbrock F., Yeh S., Vlahov N., Koennig D., Green M., Brown G., Jeyaretna S., Li Z. (2017). Perfused Three-dimensional Organotypic Culture of Human Cancer Cells for Therapeutic Evaluation. Sci. Rep..

[41] Wang Y., Kim Y.M., Langer R. (2003). In vivo degradation characteristics of poly(glycerol sebacate). J. Biomed. Mater. Res. Part A.

[42] Bettahalli N.M.S., Vicente J., Moroni L., Higuera G.A., Van Blitterswijk C.A., Wessling M., Stamatialis D.F. (2011). Integration of hollow fiber membranes improves nutrient supply in three-dimensional tissue constructs. Acta Biomater..

