# Supplementary material for: Hydrolytic Degradation and Mechanical Stability of Poly(ε-Caprolactone)/Reduced Graphene Oxide Membranes as Scaffolds for In Vitro Neural Tissue Regeneration
Source: Membranes (Basel). 2018 Mar 5;8(1):12. doi: 10.3390/membranes8010012 (PMC5872194; doi:10.3390/membranes8010012)
Supplement: Supplementary file 1 [file membranes-08-00012-s001.pdf]

membranes

MDPI

Article

Supplementary Material: Hydrolytic Degradation and Mechanical Stability of Poly(ε-caprolactone)/Reduced Graphene Oxide Membranes as Scaffolds for In Vitro Neural Tissue Regeneration

Sandra Sanchez-González [1], Nazely Diban [1,*] and Ane Urtiaga [1]

[1] Department of Chemical and Biomolecular Engineering, University of Cantabria, Avda. Los Castros s/n, 39005 Santander, Spain; sandra.sanchez@unican.es; dibann@unican.es; urtiaga@unican.es

* Correspondence: dibann@unican.es; Tel.: +34-942-206778

Videos S1 and S2 show the mechanical properties of the membranes after 12 months of degradation for PCL and PCL/rGO, respectively.

PCL resistance (t=12m).mp4

Video S1. Mechanical behaviour of PCL membranes after 12 months of hydrolytic degradation.

PCLrGO resistance (t=12m).mp4

Video S2. Mechanical behaviour of PCL/rGO membranes after 12 months of hydrolytic degradation.

Equation S1 shows 2nd order reaction kinetics of hydrolysis of polyesters, which is proportional to the concentration of water and the concentration of carboxylic bonds that are susceptible of hydrolysis,

$$\frac{dE}{dt} = k \cdot C_B \cdot C_w \cdot t \qquad \text{(S1)}$$

where $E = \rho / (N \cdot M_0)$ is the chain end concentration, $C_B = (\rho / M_0) \cdot (1 - 1/N)$ is the total bond concentration, C_w is the water concentration and k is the kinetic constant of the hydrolysis of the PCL polymer. Moreover, ρ is the density of the polymer samples, N is the degree of polymerization and M_0 is the initial monomer molecular weight.

For polyesters with large molecular weight (N>>1), carboxylic/ester polymer groups present similar probability of hydrolytic attack, and therefore the hydrolysis kinetics can be simplified to be expressed as shown below:

$$1/M_n = (1/M_{n_0}) + (1/M_0) \cdot k \cdot C_w \cdot t \qquad \text{(S2)}$$

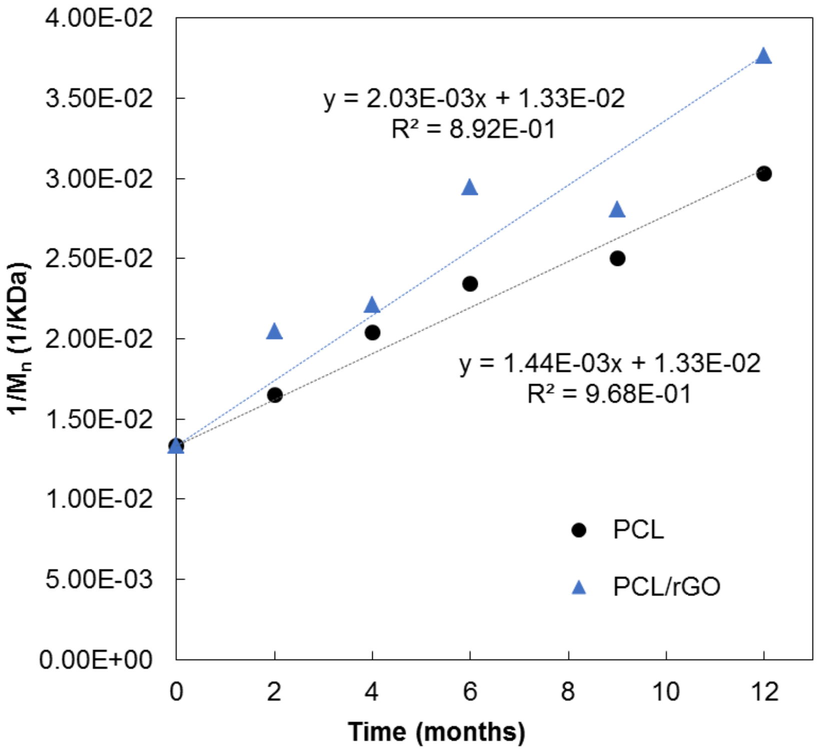

Figure S1. Kinetics of the hydrolysis of PCL and PCL/rGO membranes. Fitting of the molecular weight to the 2nd order hydrolysis kinetics (Equation S2) is depicted (dotted lines). For the present system M_0 and C_w are the same for the PCL plain membranes and the PCL/rGO material as well as Mn0. Therefore from Figure S1, it can be extracted that k(PCL)/k(PCL/rGO) = $(2.03 \times 10^{-3})/(1.44 \times 10^{-3}) \approx 1.4$.

The PBS UV-vis spectra of the initial and final PBS medium containing the PCL and PCL/rGO membranes during the degradation experiments were recorded in order to determine the fate of rGO nanoplatelets during the hydrolytic degradation of the membranes. Moreover, several 5 mg samples of PCL/rGO membranes (at 0 and 12 months of degradation) were dissolved in 10 mL of THF and centrifuged. Afterwards the undissolved rGO was qualitatively observed and compared.

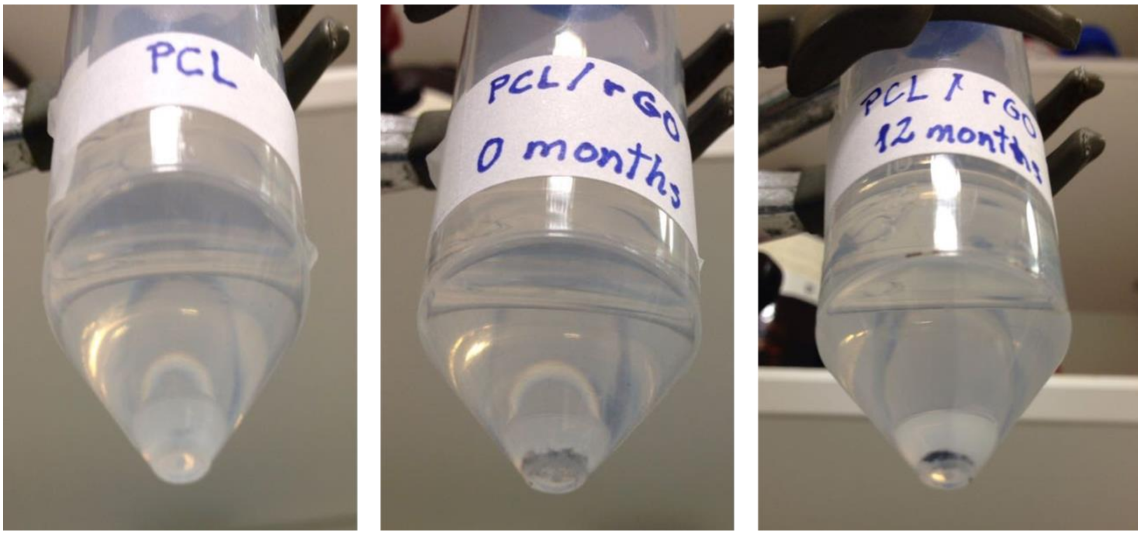

Figure S2. Photographs showing the rGO content at 0 and 12 months of degradation of the PCL/rGO membranes. (PCL is shown as control).

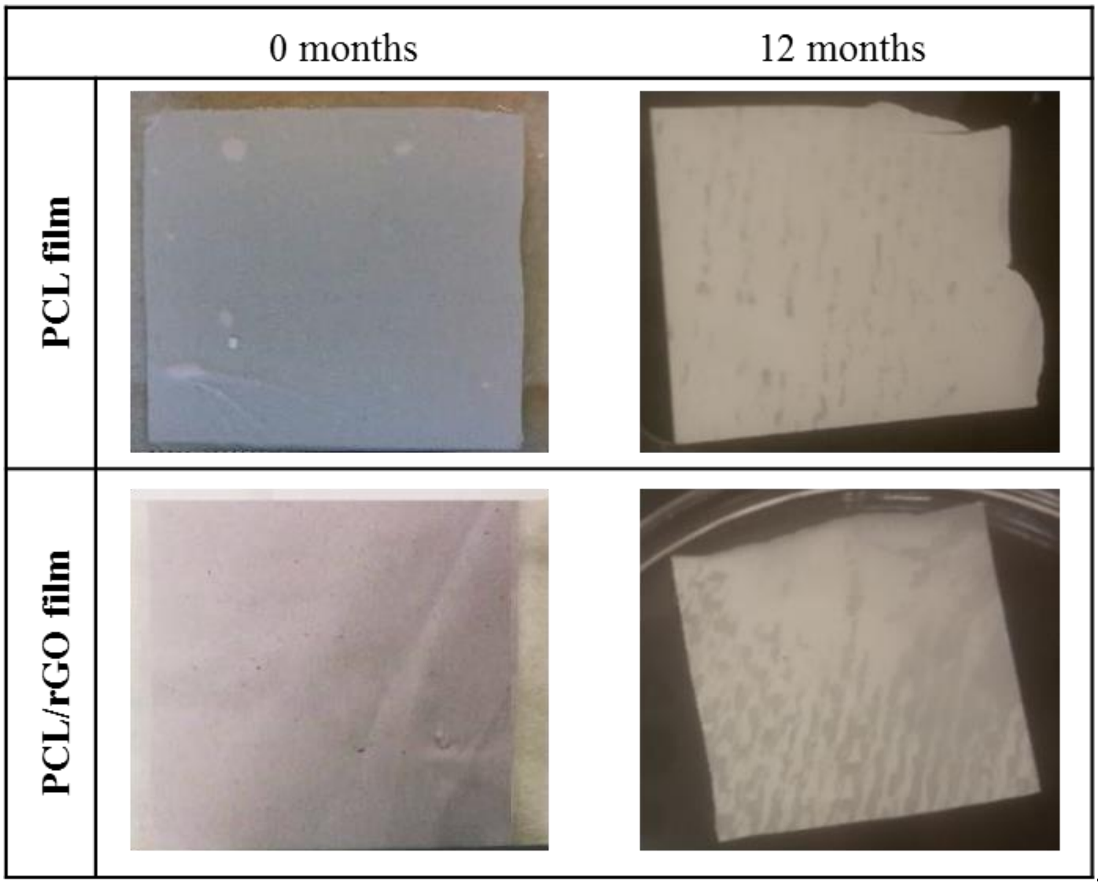

Figure S3. Photographs showing the visual aspect of the wet PCL and PCL/rGO membranes at 0 and 12 months of degradation (membrane pieces sizing 4×4 cm^2).

© 2018 by the authors. Submitted for possible open access publication under the terms and conditions of the Creative Commons Attribution (CC BY) license (http://creativecommons.org/licenses/by/4.0/).
